# Virtual Raters for Reproducible and Objective Assessments in Radiology

**DOI:** 10.1038/srep25007

**Published:** 2016-04-27

**Authors:** Jens Kleesiek, Jens Petersen, Markus Döring, Klaus Maier-Hein, Ullrich Köthe, Wolfgang Wick, Fred A. Hamprecht, Martin Bendszus, Armin Biller

**Affiliations:** 1 University of Heidelberg, Department of Neuroradiology, Heidelberg, Germany; 2 German Cancer Research Center, Junior Group Medical Image Computing, Heidelberg, Germany; 3 University of Heidelberg, HCI/IWR, Heidelberg, Germany; 4 German Cancer Research Center, Division of Radiology, Heidelberg, Germany; 5 University of Heidelberg, Department of Neurology, Heidelberg, Germany

## Abstract

Volumetric measurements in radiologic images are important for monitoring tumor growth and treatment response. To make these more reproducible and objective we introduce the concept of virtual raters (VRs). A virtual rater is obtained by combining knowledge of machine-learning algorithms trained with past annotations of multiple human raters with the instantaneous rating of one human expert. Thus, he is virtually guided by several experts. To evaluate the approach we perform experiments with multi-channel magnetic resonance imaging (MRI) data sets. Next to gross tumor volume (GTV) we also investigate subcategories like edema, contrast-enhancing and non-enhancing tumor. The first data set consists of N = 71 longitudinal follow-up scans of 15 patients suffering from glioblastoma (GB). The second data set comprises N = 30 scans of low- and high-grade gliomas. For comparison we computed Pearson Correlation, Intra-class Correlation Coefficient (ICC) and Dice score. Virtual raters always lead to an improvement w.r.t. inter- and intra-rater agreement. Comparing the 2D Response Assessment in Neuro-Oncology (RANO) measurements to the volumetric measurements of the virtual raters results in one-third of the cases in a deviating rating. Hence, we believe that our approach will have an impact on the evaluation of clinical studies as well as on routine imaging diagnostics.

The need for quantitative imaging measures to monitor tumor growth is beyond controversy^1^. In neuro-oncology, a standard procedure to account for tumor growth is based on the RANO criteria^2^. These guidelines only contain 2D surrogate measures for estimating the development of a neoplasia, even though it has been suggested^2^ and shown^3^,^4^,^5^ that a volumetric analysis is more accurate. In principle, measurements comprising not only the whole tumor volume but also the volume of subregions, like non-enhancing portions and edema, could be used to estimate tumor progression, response to treatment or to stratify patients e.g. for drug trials^1^,^6^,^7^. The emerging field of radiomics, which links imaging features with genomic and clinical data, also requires reliable segmentations, i.e. computational partitioning of images into meaningful regions^8^,^9^.

While automatic^10^,^11^,^12^ and semi-automatic methods^3^,^13^,^14^,^15^ exist, these are usually not employed in clinical routine for two major reasons. Firstly, physicians are usually pressed for time and available tools are not easily integrated in the standard clinical workflow. Secondly, most fully automatic tools are not generic enough, as they do not allow the physician to define custom normal and abnormal tissue categories. To complicate the situation, MRI is not quantitative, i.e. it does not have a standardized measurement value per voxel. Therefore, automatic methods that usually are based on machine learning algorithms and trained using a limited number of scans with certain characteristics (gray value distribution) do not necessarily work well on different types of images, e.g. acquired on a scanner of a differing vendor.

To cope with these shortcomings, we conduct this feasibility study. We propose a generic hybrid model that merges properties of semi- and fully automatic segmentation methods to create virtual raters (VRs) by combining the current assessment of a patient by a human expert with the assessments made by multiple human experts for several other patients. It has been shown that assessments vary significantly between human raters as well as between cases examined by the same rater^16^. We hypothesize that our design alleviates these effects, because the active rater can make appropriate but small changes to the aggregate opinion of multiple experts. Consequently, these virtual raters allow an objective and reproducible volumetric assessment as demonstrated by the analysis of two multi-channel MRI data sets, one containing longitudinal follow-up scans of GB, the other one a mixture of high and low grade gliomas.

## Methods

### Ethics Statement

All subjects provided written informed consent based on institutional guidelines and the local medical ethics committee (Faculty of Clinical Medicine, University of Heidelberg). All experimental protocols were approved by the aforementioned committee. For this study only pseudonymized imaging data was used, no further clinical data was evaluated. The performed data analysis was in accordance with the Declaration of Helsinki.

### Data

For experiment one longitudinal follow-up scans of 15 patients (13 male, 2 female, mean age of 55.1 y) with recurrent grade IV glioblastoma were analyzed, comprising a total of N = 71 multi-channel MRI scans (native and contrast-enhanced T1w, T2w and FLAIR images). Prior to recurrence all patients received standard of care therapy, including surgery, but no radiation therapy. Baseline scans were determined to be the first scan before de novo treatment. The median number of follow-up scans is five. Depending on radiographic and clinical diagnosis of progression by the treating neurooncologist, different experimental chemotherapy protocols became effective. These protocols included the alkylating agent Lomustine, the angiogenesis inhibitor Bevacizumab and a combination of both substances.

The sequences comprise native T1-weighted (nT1w), contrast-enhanced T1-weighted (ceT1w), T2-weighted (T2w) and T2-weighted fluid attenuated inversion recovery (FLAIR) images. The data acquisition was performed on a 3 Tesla MR-System (Magnetom Verio, Siemens Healthcare, Erlangen, Germany) with the following specifications: nT1w and ceT1w Magnetization Prepared Rapid Acquisition Gradient Echo (MPRAGE) sequence with TE = 3.4 ms and TR = 1740 ms and a voxel resolution of 1 × 1 × 1 mm; T2w Spin Echo (SE) imaging with TE = 85 ms and TR = 5520 ms and a voxel size of 0.63 × 0.63 mm, slice spacing 5 mm; FLAIR TSE imaging with TE = 135 ms and TR = 8500 ms and a voxel size of 0.94 × 0.94 mm, slice spacing of 5 mm.

The second experiment was conducted using data (N = 30) of the 2013 Brain Tumor Segmentation (BraTS) challenge. This publicly available data set^17^ contains scans of high and low grade gliomas, each comprising the aforementioned four MRI channels. For details please see^11^.

### Data Preprocessing

For experiment one the data was preprocessed as follows. First, a N3 bias field correction was applied to nT1w, ceT1w and T2w images, but not to the FLAIR images (in preliminary experiments a bias field correction of the FLAIR images did not prove to be advantageous). Next, all four modalities were resampled to a resolution of 1 × 1 × 1 mm^3^. For bias field correction and resampling the FreeSurfer^18^ tools *nu_correct* and *mri_convert* were employed. Further, we performed a longitudinal registration for each patient based on their native T1w images, using the FreeSurfer tool *mri_robust_template*^19^. We used FSL-BET^20^ to compute a brain mask for this template. Next, all channels of each time point were intra-individually registered to the respective nT1w volume using a 6-DOF linear registration as implemented in FSL-FLIRT^21^. The resulting transformation matrices were each concatenated with the nT1w transformation matrix of that time point (as obtained during the longitudinal registration) and then used to transform all channels to a common space. Now the previously acquired brain mask was applied to all scans. A senior neuroradiology resident visually confirmed the accuracy of the registration and the brain extraction.

As the BraTS data used for experiment two was acquired on scanners from different vendors, it was processed differently. The downloadable data is already brain extracted, co-registered and resampled to a resolution of 1 × 1 × 1 mm^3^. After a N3 bias field correction of the nT1w, ceT1w and T2w images, we employed histogram normalization as implemented by the *HistogramMatching* routine of 3D-Slicer^22^. For reference images we used the four different modalities of an arbitrary data set (HG0001). To exclude the background during matching, all voxels whose grayscale values were smaller than the mean grayscale value were excluded. Further we performed normalization as proposed in Kleesiek, *et al.*^23^.

For both experiments we computed a subtraction of the contrast-enhanced and native T1w image, following^24^. Further, within the ilastik framework we computed the following features: Gaussian Smoothing (scale 1.0 and 3.5), Laplacian of Gaussian (scale 1.0 and 3.5), Difference of Gaussians (scale 1.0 and 3.5), Gaussian Gradient Magnitude (scale 1.0 and 3.5), the Structure Tensor Eigenvalues (scale 1.0 and 3.5) and the Hessian of Gaussian Eigenvalues (scale 1.0 and 3.5).

Note, the described process leads to 4D (experiment 2) or 5D (experiment 1) data for each patient: [t]xyzc, where *t* denotes time, *x,y,z* corresponds to coordinates in space and *c* to different channels. The channels comprise the raw data as well as the computed features, totaling in our case 65 entries. When a human rater annotates the data using the MRIVolumetry workflow, the machine learning algorithm simultaneously analyzes this high-dimensional vector of values and is able to extract linear and non-linear relations for learning. This explains the effectiveness of this approach.

### Volumetry

**Figure 1:**
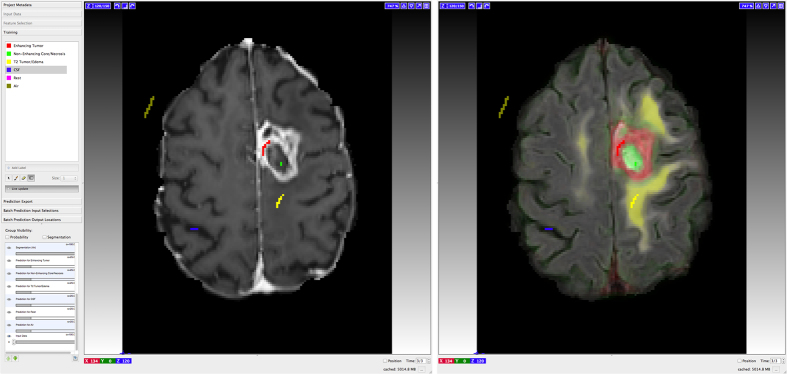
Graphical user interface of the presented software tool. The left side displays a contrast-enhanced T1w image of a GB patient; the corresponding FLAIR image with an overlay of predicted class probabilities is shown on the right. Annotations (Scribbles) are displayed in different colors: contrast enhancement (red), non-enhancing tumor (green), edema (yellow), CSF (blue) and air (dark yellow). For a demonstration please see Video 1.

For segmentation we developed a custom MRIVolumetry workflow (Fig. 1) based on the PixelClassification workflow of the ilastik project^25^. It utilizes ten random forests^26^ with ten trees each that are trained in parallel and eventually merge into a single forest. Processing of the pseudo-probabilities (obtained from the random forest predictions) comprises a Gaussian smoothing with σ = 1.2 voxels, followed by an argmax computation and removal of connected components smaller than 1000 voxels. The software is available for all major platforms (Windows, Linux and Mac).

In contrast to existing programs the tool allows users to freely define tissue categories (classes) used for segmentation and to work with multi-modal data containing an arbitrary number of channels (only limited by the RAM of the computer). Domain experts can interactively annotate data with scribbles while the underlying machine-learning algorithm captures this knowledge and is able to make class predictions for the entire visible image in close to real-time. Optionally, the expert can be pointed to regions with a high uncertainty and then supply missing annotations. Once sufficiently trained, the tool can be used in batch mode to automatically process huge amounts of data. For a demonstration please see Video 1.

### Experiments

**Figure 2:**
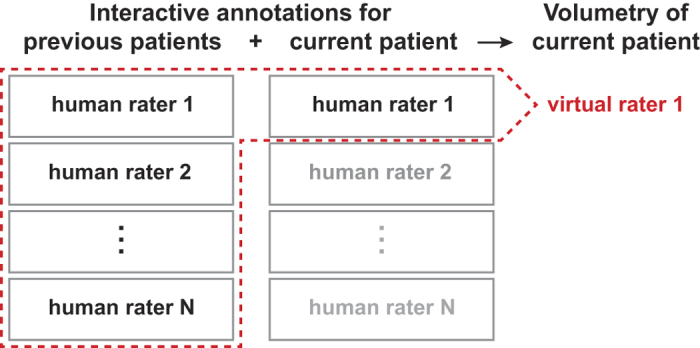
Virtual Rater Concept. Interactive human rater annotations of all previous patients are combined with the interactive annotations of a single rater for the current patient. This pooled information is used to train an independent machine-learning algorithm for the current patient. This creates a virtual rater companion for every human rater and is exemplarily shown for virtual rater 1.

For experiment one we defined six different categories: *Contrast-Enhancing Tumor*, *Non-Enhancing Tumor*, *Edema*, *Cerebrospinal Fluid*, *Rest* and *Air*. Two experienced neuroradiologists were instructed to only label regions if they are absolutely certain that these voxels belong to the respective category. The workflow is designed so that the expert can easily toggle between all channels. Labels can be placed in any of the channels. Because the data is co-registered a single label comprises all available channels. Time points were segmented separately and consecutively. Each expert interactively segmented the data twice at different times (run a and b). We timed this sparse labeling process for the second run of rater 1 to compare it to the time necessary to perform the standard clinical RANO measurements. Further, we generated a virtual rater for each expert (Fig. 2). For this purpose we held back the longitudinal data set of one patient and trained the algorithm offline with all the remaining labels of both raters and both runs plus the combined label sets of rater 1 (or rater 2) for the left out data set. Computing the class predictions for rater 1 (or rater 2) yielded the virtual rater 1 (2). This was repeated for all patients. Based on the segmentations of the human and virtual raters we computed the volume of tumor regions for comparison. In addition, we made 2D RANO measurements (product of orthogonal diameters).

To confirm that the segmentation quality of a virtual rater is similar to that of a human rater, we performed a cross-validation akin to experiment 2 (see below). For each dataset we combined the four available segmentations (two raters at two time points) using the STAPLE algorithm^27^, which uses expectation-maximization to compute a probabilistic estimate of the underlying true segmentation, and treated the result as a reference segmentation. We then compared the single human raters and the virtual raters with this reference for each dataset and averaged the results.

For experiment two, manual segmentations of four different expert raters are publicly available. These contain five different classes: *Normal*, *Necrosis*, *Edema*, *Non-Enhancing* and *Contrast-Enhancing Tumor*. Again we combined the human segmentations using the STAPLE algorithm^27^ and used this result to train our algorithm. We computed automatic segmentations using leave-one-out cross-validation and created, akin to the first experiment, virtual raters (Fig. 2). Because only dense annotations are available, we randomly picked up to 200 training samples from each tumor class in addition to 1000 samples from normal tissue.

To model a more realistic scenario where annotations for a datasets from all/the same experts might not always be available, we repeated the second experiment and allowed each segmentation instance (single expert segmentation of an image volume) to only be present with a probability P. Using the STAPLE algorithm we again merged the remaining segmentations for a given dataset (or left it out if none remained) and proceeded as outlined above. We performed this experiment with P = 0.75, P = 0.5 and P = 0.25, so that on average 3, 2 and 1, respectively, segmentations remained per dataset for training.

For both experiments the category gross tumor volume (GTV) was obtained by merging all tumor-related classes.

### Analysis

Statistical analysis war performed using R^28^ and custom python scripts. For estimating reproducibility, the intra-class correlation coefficient (ICC) was calculated using the R package IRR^29^. The intra-rater consistency was computed according to case one of McGraw and Wong^30^.

Combination of the p-values of the Pearson correlations for the longitudinal volumetric measurements was performed using Fisher’s method^31^.

For comparison of the segmentations we calculated the Dice coefficient^32^, a widespread measure commonly used in the field. The ratio is defined as twice the size of the intersection of the two masks normalized by the sum of their combined sizes. The coefficient takes values in the range [0.0, 1.0], where 1.0 is obtained for identical segmentations.

## Results

### Experiment 1 – Longitudinal follow-up of GB patients

Volumetry of rater 1 took on average 3 min. and 26 s per examination (Table S1), which is, according to our experience, slightly faster as the time usually taken to perform RANO measurements but comparable to the time reported by other semi-automatic methods^13^,^14^. The more time points a longitudinal examination contains, the higher the resulting time saving. This is reflected by a negative correlation (Pearson correlation r = −0.72, p = 0.003) between duration and number of longitudinal examinations (cf. Fig. S1). Details on the label statistics are summarized in the supplement.

**Figure 3:**
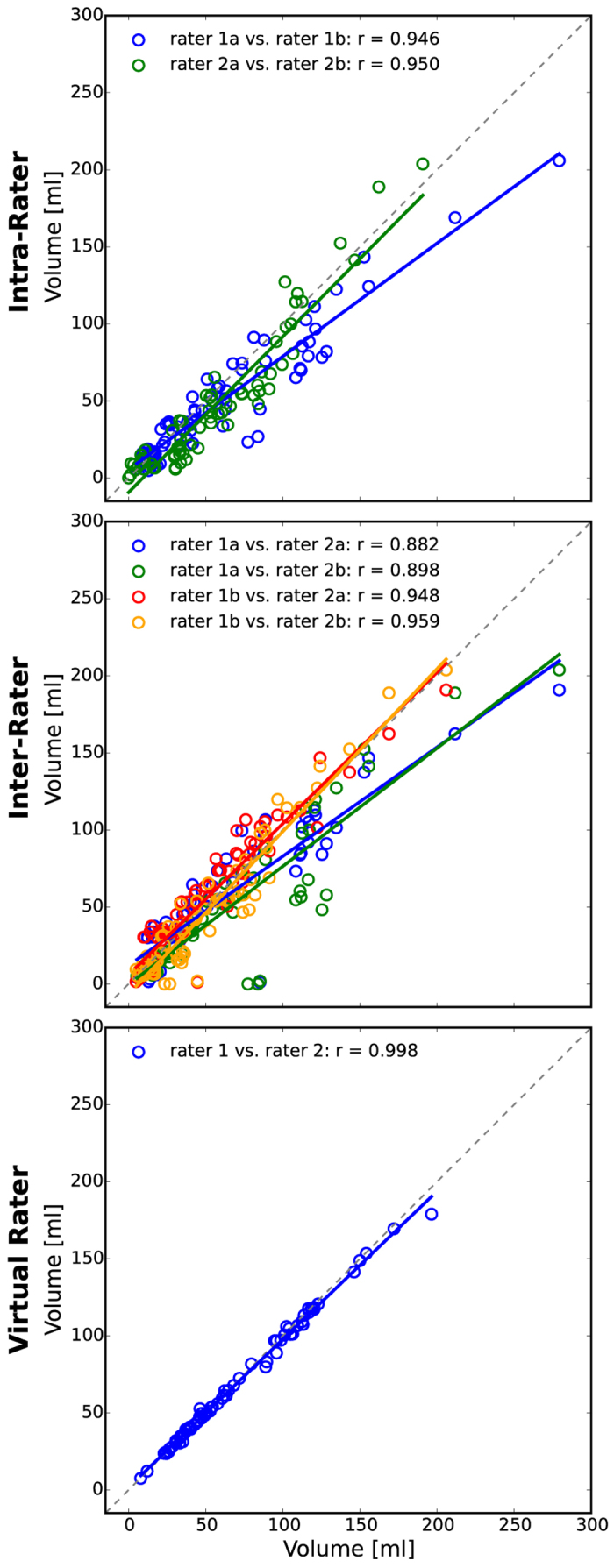
GTV scatter plots showing intra-, inter- and virtual-rater Pearson correlation for experiment 1 (N = 71 MRI scans). All results are significant (p ≪ 0.0001). The correlation for the virtual raters is higher than for the human experts. For scatter plots showing edema, contrast-enhancing and non-enhancing tumor categories please see supplement.

To visualize the inter-rater and intra-rater accordance we use scatter plots for the GTV (Fig. 3), the edema (Fig. S2a), the enhancing (Fig. S2b) and non-enhancing volumes (Fig. S2c). As expected, the lowest intra-rater correlation can be found for the non-enhancing volume, as this class is typically most prone to rater subjectivity. The lowest inter-rater correlation can be found for the edema class. For instance, here the differentiation to gliotic alterations is not always feasible without ambiguity. Still, we yield an inter-rater Pearson correlation of at least r = 0.79 (p ≪ 0.0001). For the GTV the minimum inter-rater correlation is r = 0.88 (p ≪ 0.0001). For all four categories the interactive virtual raters lead to a drastic improvement of correlation. The Pearson correlation for the two virtual raters is r = 0.969 (p ≪ 0.0001) for the non-enhancing class; the other three categories show a correlation of at least r = 0.995. Further, we estimated the repeatability of the GTV intra-rater segmentations using ICC. We obtain for rater 1 ICC(1) = 0.89 (p ≪ 0.0001) and for rater 2 ICC(1) = 0.93 (p ≪ 0.0001). The ICC for the virtual rater is significantly higher (ICC = 0.997, p ≪ 0.0001).

**Figure 4:**
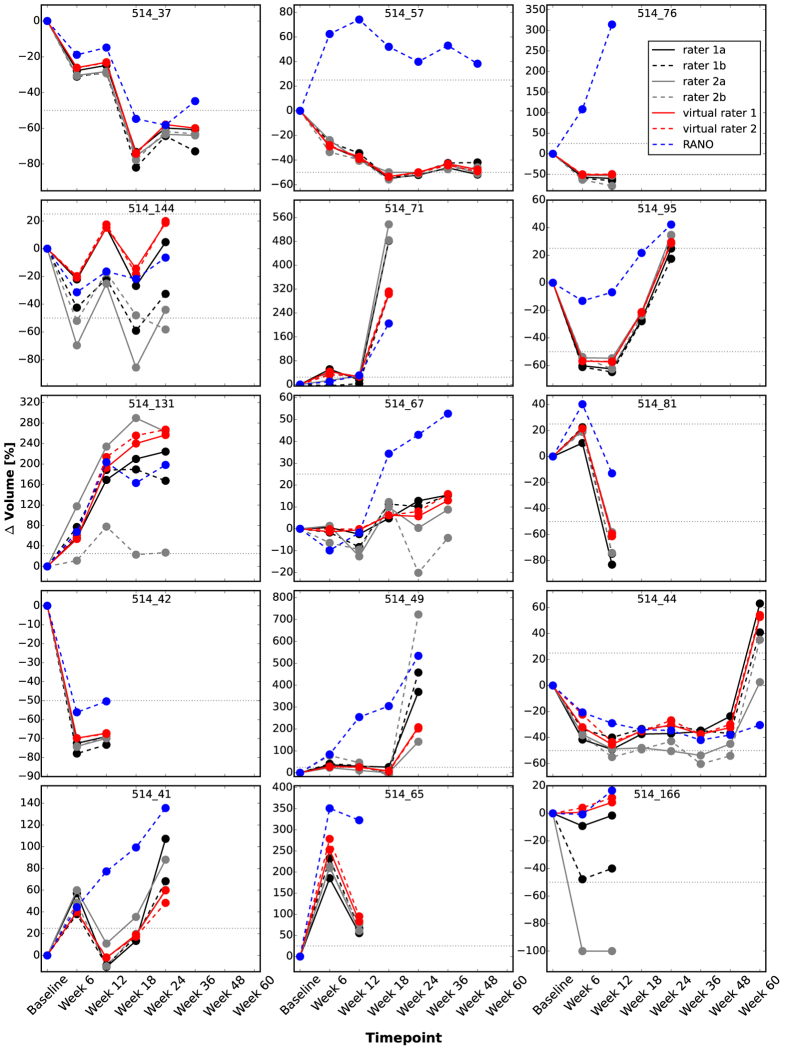
Relative longitudinal changes of GTV for 15 patients suffering from GB. The two human raters interactively segmented the tumor images twice (two independent sessions a and b). The virtual raters show a higher agreement amongst each other but in principle meet the assessments of the human experts. By comparing the volumetric analysis to 2D RANO measurements discrepancies become visible. Dotted horizontal lines indicate the RANO cutoffs: 25% increase is defined as *progression*, a 50% decrease as *partial response*.

Considering the change of GTV (dGTV) w.r.t. the baseline examination, an average correlation of r = 0.94 (p ≪ 0.0001) is calculated for the longitudinal time series of the human raters. Again, the virtual raters yield a higher correlation (r = 0.995, p ≪ 0.0001). The change of GTV is a more meaningful measure than GTV in this setting, because it defines progression and response as in the RANO criteria (see below), which are most widely used to come up with a therapeutic decision. We also compared the dGTV time courses qualitatively. Figure 4 shows the dGTV for all patients; the absolute GTV and the volume for different tumor subregions are depicted in supplementary Figures S3a–d. In general, these plots confirm the correlation results and show a good intra- and inter-rater agreement. In comparison to the human raters, the results of the virtual raters are more similar. For instance, investigating the volumetric differences between the two human raters for the segmentation of patient *514_166* revealed that rater 1 interpreted the widespread T2-elongations flanking the tumor as edema whereas rater 2 assumed gliotic alterations as the underlying cause. Usually, the biggest deviations between the human raters and the virtual raters are found for the non-enhancing tumor portions (e.g. *514_131*, Fig. S3d). Note, these regions often account for only a small fraction of the total tumor volume.

In addition to the tumor volumetry, we also performed 2D RANO measurements for the 15 patients. Remarkably, applying the RANO guidelines (25% increase defines *progression*, 50% decrease defines a *partial response*, dashed gray lines) to the volumetric measurements would have let in one third of the cases to a different rating (Fig. 4), even if different ranges (40% increase, 65% decrease), previously proposed for volumetric measures, had been employed^13^,^33^.

**Figure 5:**
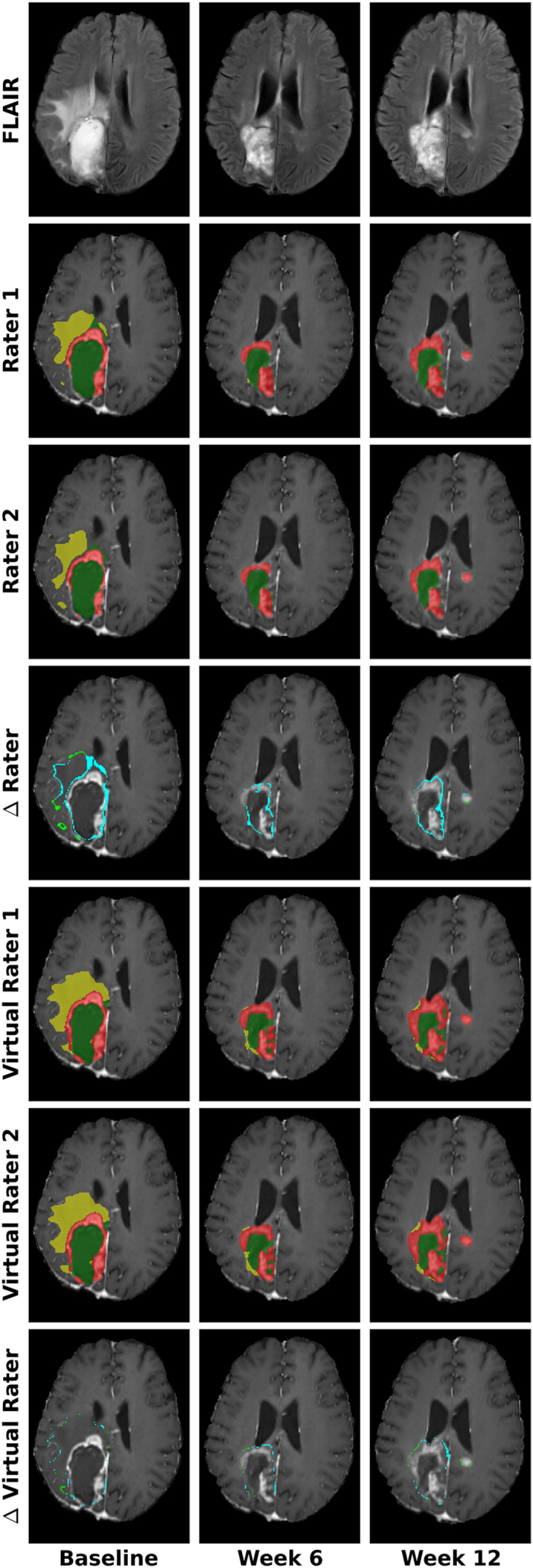
Comparison of differences in tumor segmentations obtained by human experts (session a) and virtual raters. Virtual raters show a higher agreement, exemplarily shown for the longitudinal follow-up examination of patient *514_42*. Top row displays the FLAIR images for the three time points. The other images depict overlays of the segmentations, or their differences, on corresponding contrast-enhanced T1w images. For comparison additional (cyan) and missing (light green) voxels are emphasized. Note, these difference-voxels might have an attenuated effect on the total volume, because they partially cancel each other out during summation. Edema (yellow), contrast-enhancing (red) and non-enhancing (green) tumor regions.

Figure 5 depicts corresponding axial slices of patient *514_42* for different time points. Comparing the differences of the segmentation of the human raters versus the virtual raters again emphasizes the higher agreement of the latter. It has to be pointed out that the differences of the segmentations have an attenuated effect on the total volume, as additional (cyan) and missing (light green) voxels between the two segmentations partially cancel out each other during summation.

Cross-validation comparing the single human raters and virtual raters against a reference obtained by merging the four available segmentations for a given dataset yielded mean Dice scores of 0.635 (0.191) for the human raters and 0.636 (0.166) for the virtual raters for the GTV segmentation. According to Welch’s two sample t-test there is no statistical difference between the groups (t(320) = −0.047, p = 0.96). Detailed results for all subclasses can be found in Table S3.

### Experiment 2 – Evaluation on BraTS data

**Figure 6:**
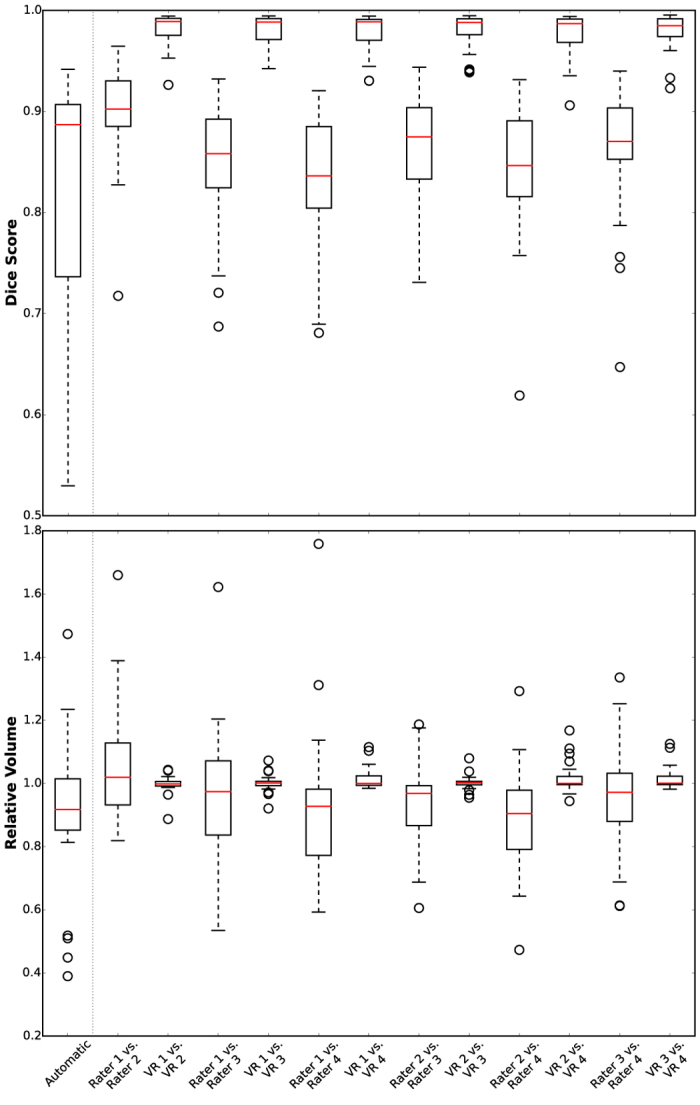
Dice Score (top) and relative volumes (bottom) for the BraTS data. The inter-rater differences for the gross tumor segmentations of the four human raters and their virtual counterparts (VR) are shown. Using our algorithm in fully automatic mode (leave-one-out cross-validation), we achieve a median value comparable to the inter-rater differences. However, the proposed virtual raters (VR) lead to a boost in agreement and display less variance compared to human experts. Median is displayed in boxplots; circles represent outliers outside 1.5 times the interquartile range of the upper and lower quartile, respectively.

In Fig. 6 the inter-rater differences between the gross tumor segmentations within the four human raters and their virtual counterparts are shown. Comparing human inter-rater Dice score to the virtual inter-rater scores shows significant improvements (Fig. 6 top; for statistics please see supplement Table S4); this is also reflected by a drastic reduction in variance. The same effect is also reflected by the relative volume changes, i.e. the ratio of volumes computed from two corresponding segmentations, within the raters (Fig. 6 bottom), which shows less variance and a median value close to 1.0 for the virtual raters. For the tumor volume human raters achieve an ICC(1) = 0.96 (p ≪ 0.0001), whereas ICC(1) = 1.0 (p ≪ 0.0001) is reached by the virtual raters. The results of our algorithm in fully automatic mode yield a median Dice score comparable to human inter-rater scores (Fig. 6).

The average Dice score for GTV segmentation of all virtual rater pairs, i.e. the mean of the six VRx/VRy comparisons in Fig. 6, is 0.963 (0.043). When each expert segmentation is only present with a probability P = 0.75, the mean Dice score is 0.958 (0.053); for P = 0.5 it is 0.956 (0.060) and for P = 0.25 a value of 0.949 (0.078) is obtained. The human raters achieve a mean Dice score of 0.825 (0.069). Results for all subclasses are summarized in Table S5.

We also computed the Dice scores for GTV segmentation of all raters with the corresponding reference segmentation, which is the merged segmentation of all four experts. The mean score of the virtual raters is 0.817 (0.097) for P = 1.0, 0.805 (0.109) for P = 0.75, 0.809 (0.108) for P = 0.5 and 0.788 (0.168) for P = 0.25. The human raters achieved a mean score of 0.899 (0.047).

**Figure 7:**
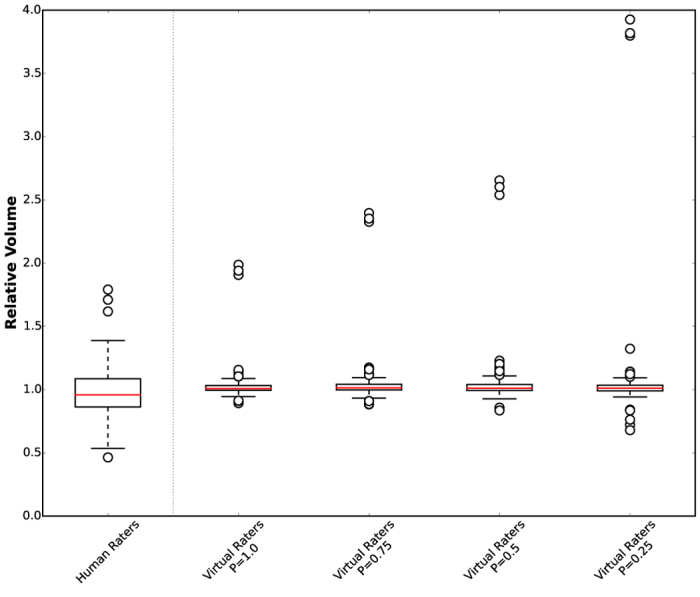
Mean relative volumes for the BraTS data. The averaged relative volumes of all human raters, i.e. HR1 vs. HR2, HR1 vs. HR3 etc. for a given dataset, and all virtual raters for different probabilities P are shown. The distance of the outliers of the virtual raters increases as the number of available expert decreases. Even for P = 0.25, where on average only one expert annotation per dataset is available, the virtual raters demonstrate a smaller interquartile range than human raters; circles represent outliers outside 1.5 times the interquartile range of the upper and lower quartile, respectively.

Next to the Dice score, we also investigate the mean relative volumes for the BraTS data if only some experts provide annotations that can be used for training (Fig. 7). Looking at the relative volume the VRs demonstrate again less variance. For P = 0.5 the variance approximately matches that of the human raters. Further, the more expert annotations are available on average for training, the less severe are the outliers that can be found. Results for all subclasses are summarized in Table S6.

## Discussion

In this feasibility study we introduce the notion of virtual raters that can be obtained based on annotations of human (expert) raters. In experiments with different multi-channel MRI data of high and low grade brain tumors we demonstrate that the approach leads to a high reproducibility and thus objectivity of volumetric measurements (Figs 3, 4, 5, 6, 7).

Previously, it has been shown that automatic methods achieve a performance comparable to human experts^11^ and that an increasing automation of imaging measurements displays less variability compared to manual techniques^5^,^34^. Here we elucidate an idea that improves on these results and makes these methods ready to find their way into clinical routine. The proposed method represents a continuous hybrid between semi- and fully automatic segmentation methods and is able to combine their strengths. In contrast to existing semi-automatic methods^3^,^13^,^14^,^15^ the knowledge gained during annotation of single data sets is conserved and is subsequently used to rate images of novel patients or other time points of the same patient. Using our tool in patient-specific semi-automatic mode, without taking prior knowledge into account, leads to ICC values comparable to previously published results^35^. Introducing the concept of virtual raters (Fig. 2) improves the results drastically without causing increased human efforts.

If desired, the acquired expertise can be employed in fully automatic mode to unseen data (cf. Fig. 6). However, we propose to combine the previously acquired knowledge with annotations of the patient currently under investigation. This leads to clear advantages in comparison to automatic methods, as the grey value distribution of the scan at hand is taken into account for the delineation of the structure. For example, it has been shown that the Gadolinium contrast enhancement is non-linear^36^. Thus, given a limited number of training samples, it is not surprising that fully automatic methods start to falter when dealing with this subcategory^11^.

In contrast to existing methods, arbitrary tissue categories can be defined for the interactive workflow and the generic procedure is not limited to MRI scans of brain tumors but also can be employed to other modalities (e.g. Computer Tomography), other diseases (e.g. stroke, cerebral hemorrhage or inflammatory disease) and other body regions.

Intra-axial brain tumors rather invade the surrounding tissue opposed to merely displacing it. This usually leads to irregularly shaped and ambiguous borders. We do not claim that the “correct” border, if something like this should exist, is found by our method. Instead, a reproducible and consistent decision is learned (Fig. 5), guided by the statistics of the data in conjunction with the expert’s knowledge.

Figure 5 already illustrates that the segmentation quality of the virtual raters is comparable to that of the human raters. Comparison of the virtual raters and the single human raters against a reference segmentation that was created by merging the four available segmentations for a given dataset resulted in two important observations. A mean Dice score of 0.64 obtained for the human raters’ GTV segmentation is indicative of the low inter- and intra-rater agreement. Virtual raters yield a score that is statistically not different from that of the human experts. This demonstrates that the segmentation quality is comparable and substantiates our claim that VRs lead to more reproducible results. Especially, when comparing the volume change, which is commonly used as the basis for therapeutic decisions, it is important that at each longitudinal examination the decision if a region is part of the tumor or not is based on the same criteria, i.e. the gray values of the voxels. This is given by our approach, as the trained model leads to consistent decisions that are only influenced to minor extent by the current rating of the human expert. Even though the human rater can ‘overwrite’ the decision of the machine learning algorithm by placing a sufficient number of labels.

Human raters and VRs achieve comparable results. However, the *real* ground truth is not known and even the opinions of two experts can vary profoundly. Our results demonstrate that it would be ideal to combine the assessment of more than two experts, for which the proposed method inherently offers the possibility. But it is not necessary that the underlying expert annotations for training the virtual raters come from the same experts (cf. Fig. 7).

The need for reproducible volumetric tumor measurements is further manifested by the fact that our approach would have let to a different response assessment – in comparison to the RANO assessments – in one third of the cases. It is well established that volumetry is more accurate than two-dimensional surrogate measures^3^,^4^,^5^ and our method allows radiologists to perform it with little time and effort.

The reported annotation times for our first experiment are within the range of other semi-automatic methods^13^,^14^. In our experiment the raters where instructed to place labels i) in regions where they are absolutely certain for a given category and ii) continue until satisfaction. Loosening the second constraint will further decrease the labeling times and make the method applicable for clinical routine. As shown by Goetz, *et al.*^37^ sparse but confident annotations suffice to obtain good segmentations.

In conclusion, we propose to collect simple annotations (scribbles) during routine imaging diagnostics and combine these labels to train a virtual rater. This can be accomplished with minimal time effort and will lead to more reproducible and objective ratings.

## Additional Information

**How to cite this article**: Kleesiek, J. *et al.* Virtual Raters for Reproducible and Objective Assessments in Radiology. *Sci. Rep.* **6**, 25007; doi: 10.1038/srep25007 (2016).

## Supplementary Material

Supplementary Video 1

Supplementary Information
