# Supplementary material for: Virtual Raters for Reproducible and Objective Assessments in Radiology
Source: Sci Rep. 2016 Apr 27;6:25007. doi: 10.1038/srep25007 (PMC4846987; doi:10.1038/srep25007)
Supplement: Supplementary Information [file srep25007-s2.pdf]

Supplemental Material for

Virtual Raters for Reproducible and Objective Assessments in Radiology

Authors: Dr. Dr. Jens Kleesiek, MD, PhD
Jens Petersen
Markus Döring
Dr. Klaus Maier-Hein, PhD
Dr. Ullrich Köthe, PhD
Prof. Wolfgang Wick, MD
Prof. Fred A. Hamprecht, PhD
Prof. Martin Bendszus, MD
Dr. Armin Biller, MD

Supplementary Results

Label Statistics

In comparison to rater 1, rater 2 marked fewer voxels during the interactive labeling process. Rater 1 applied on average 12 brush strokes per volume with an average length of 9.9 voxels. Rater 2 used on average more brush strokes (20). However, these had a shorter length on average (4.2 voxels). Details are summarized in Table S2. Brush strokes were estimated using connected components.

Supplementary Figures

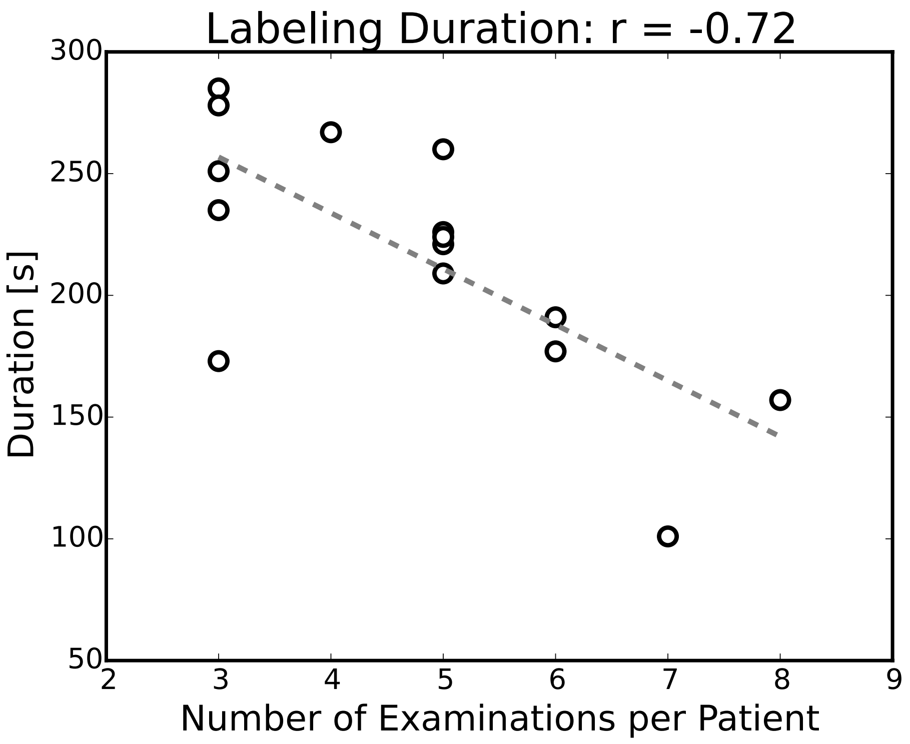

Figure S1: Relationship of annotation duration and number of follow-up scans. The more time points a 5D image data set of a patient contains, the less annotation time is needed on average during the proposed interactive workflow.

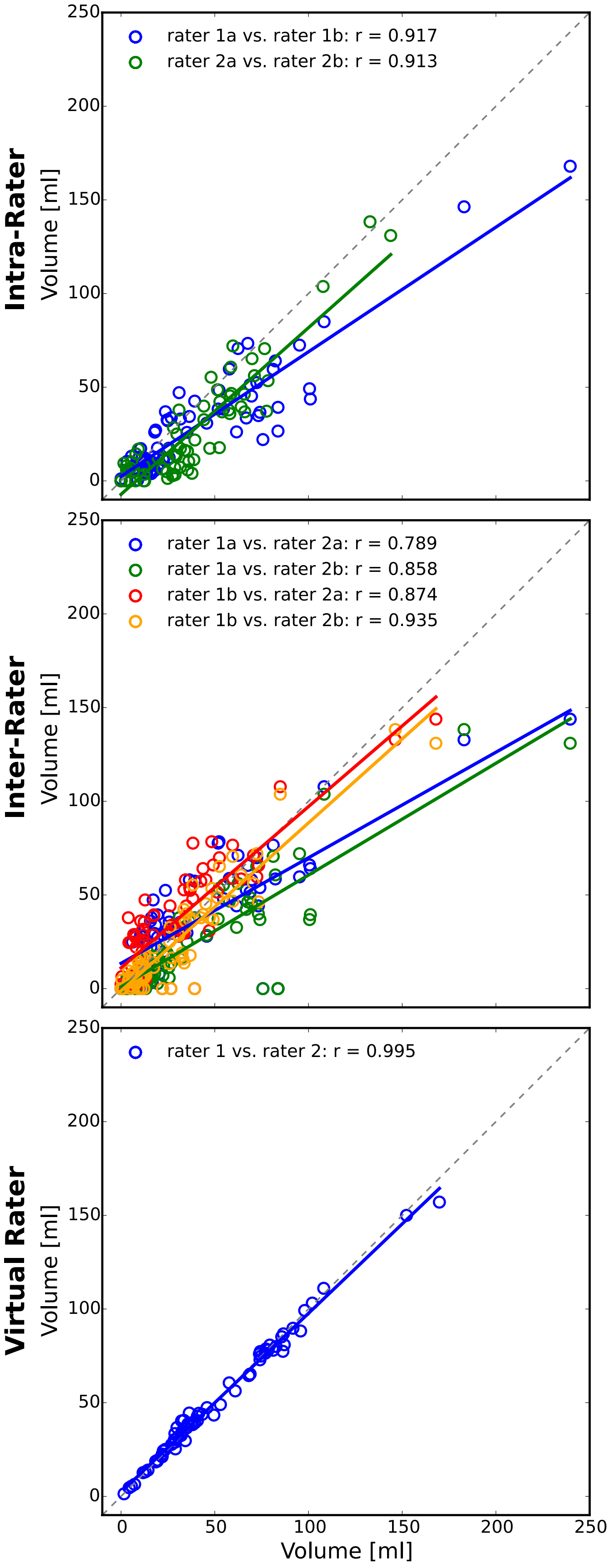

Figure S2a: Scatter plots showing intra-, inter- and virtual-rater Pearson correlation for the tumor edema category (N=71 MRI scans). All results are significant ($p<<0.0001$). The correlation for the virtual raters is higher than for the human experts.

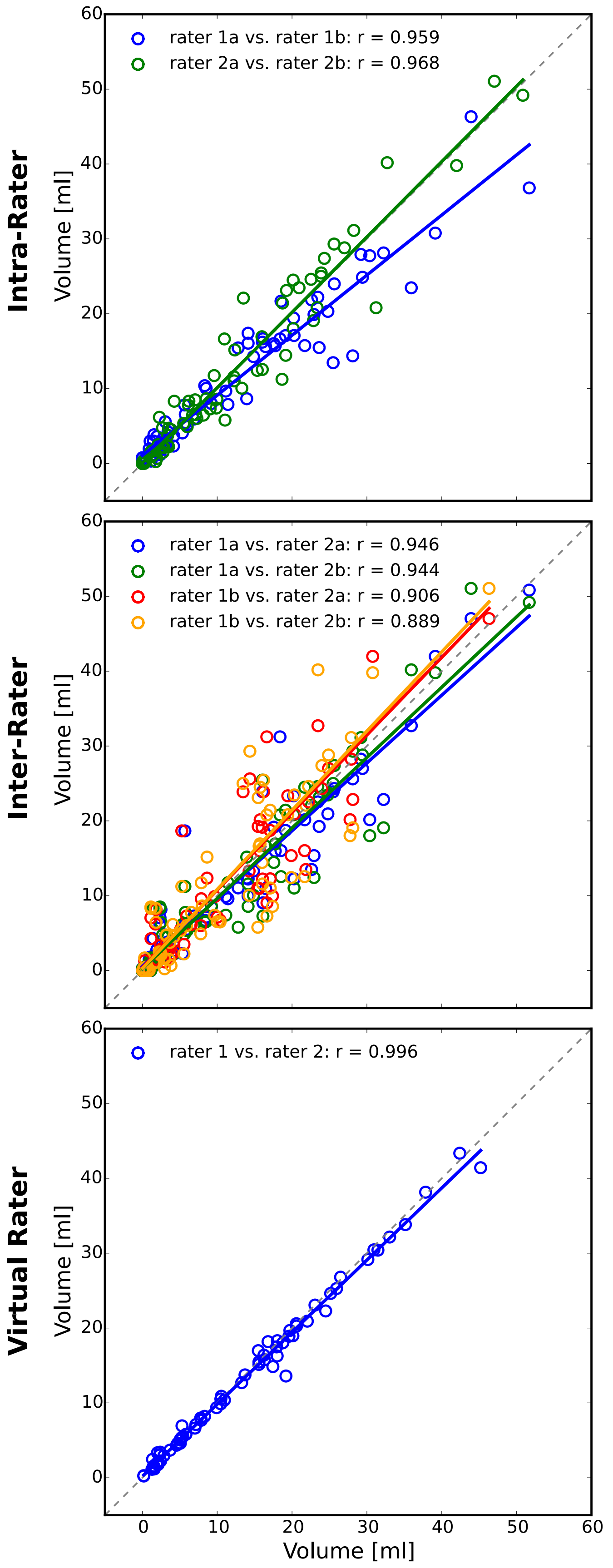

Figure S2b: Scatter plots showing intra-, inter- and virtual-rater Pearson correlation for the contrast-enhancing tumor category (N=71 MRI scans). All results are significant ($p<<0.0001$). The correlation for the virtual raters is higher than for the human experts.

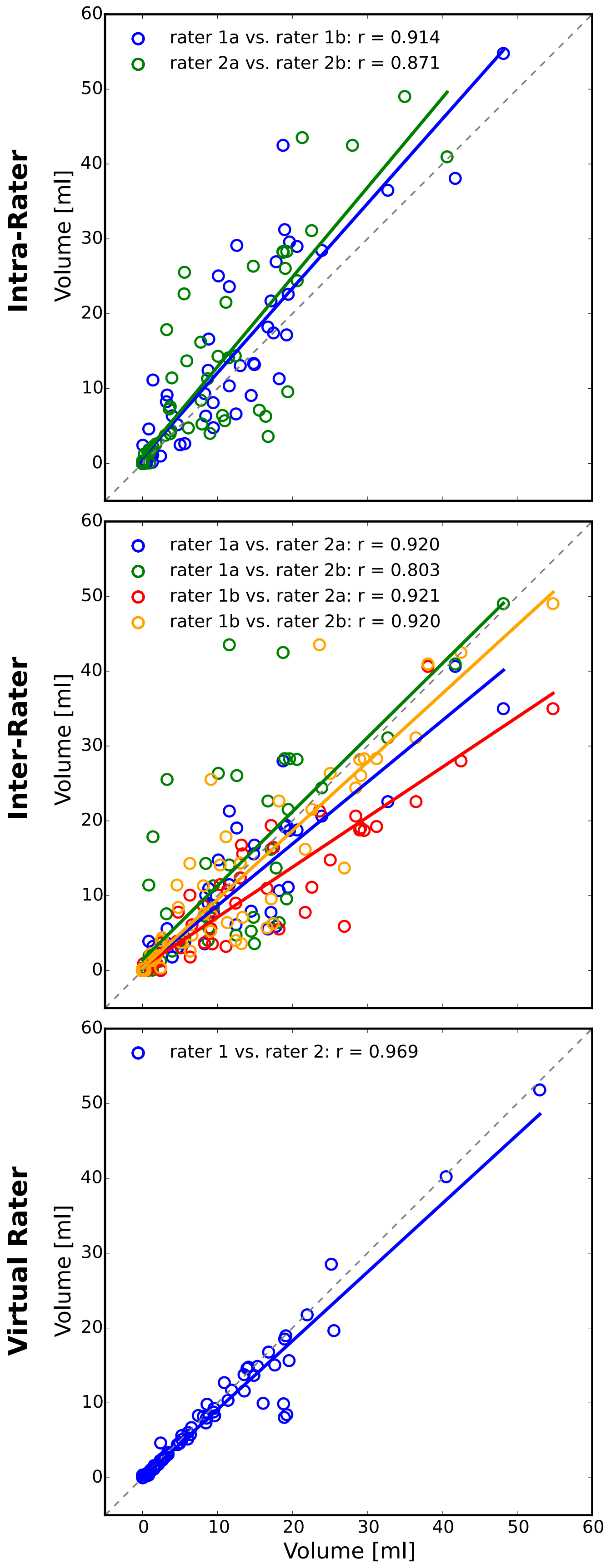

Figure S2c: Scatter plots showing intra-, inter- and virtual-rater Pearson correlation for the non-enhancing tumor category (N=71 MRI scans). All results are significant (p<<0.0001). The correlation for the virtual raters is higher than for the human experts.

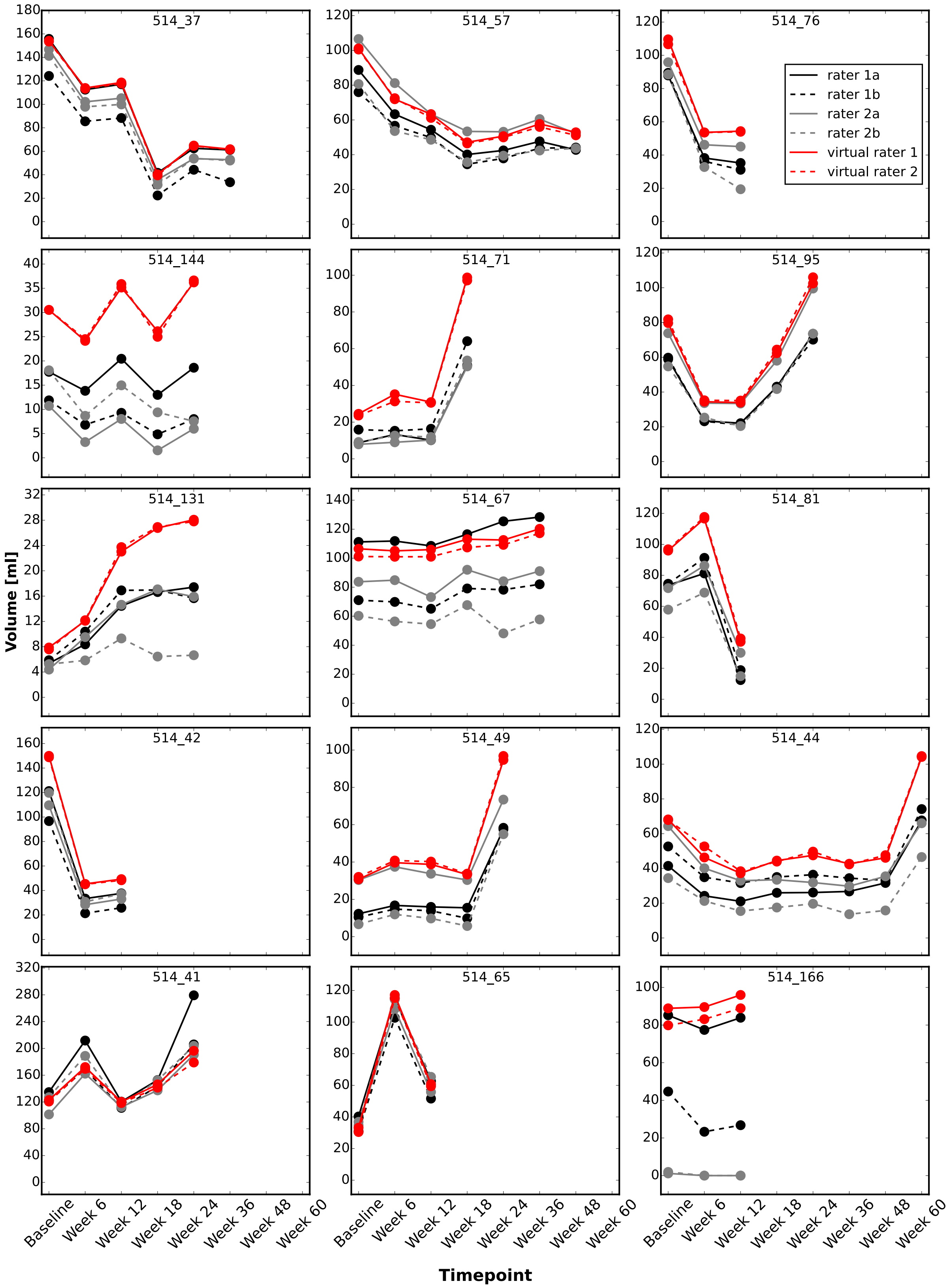

Figure S3a: Longitudinal GTV for 15 patients suffering from GB. The two human raters interactively segmented the tumor images twice (two independent sessions a and b). The virtual raters show a higher agreement amongst each other but in principle meet the assessments of the human experts.

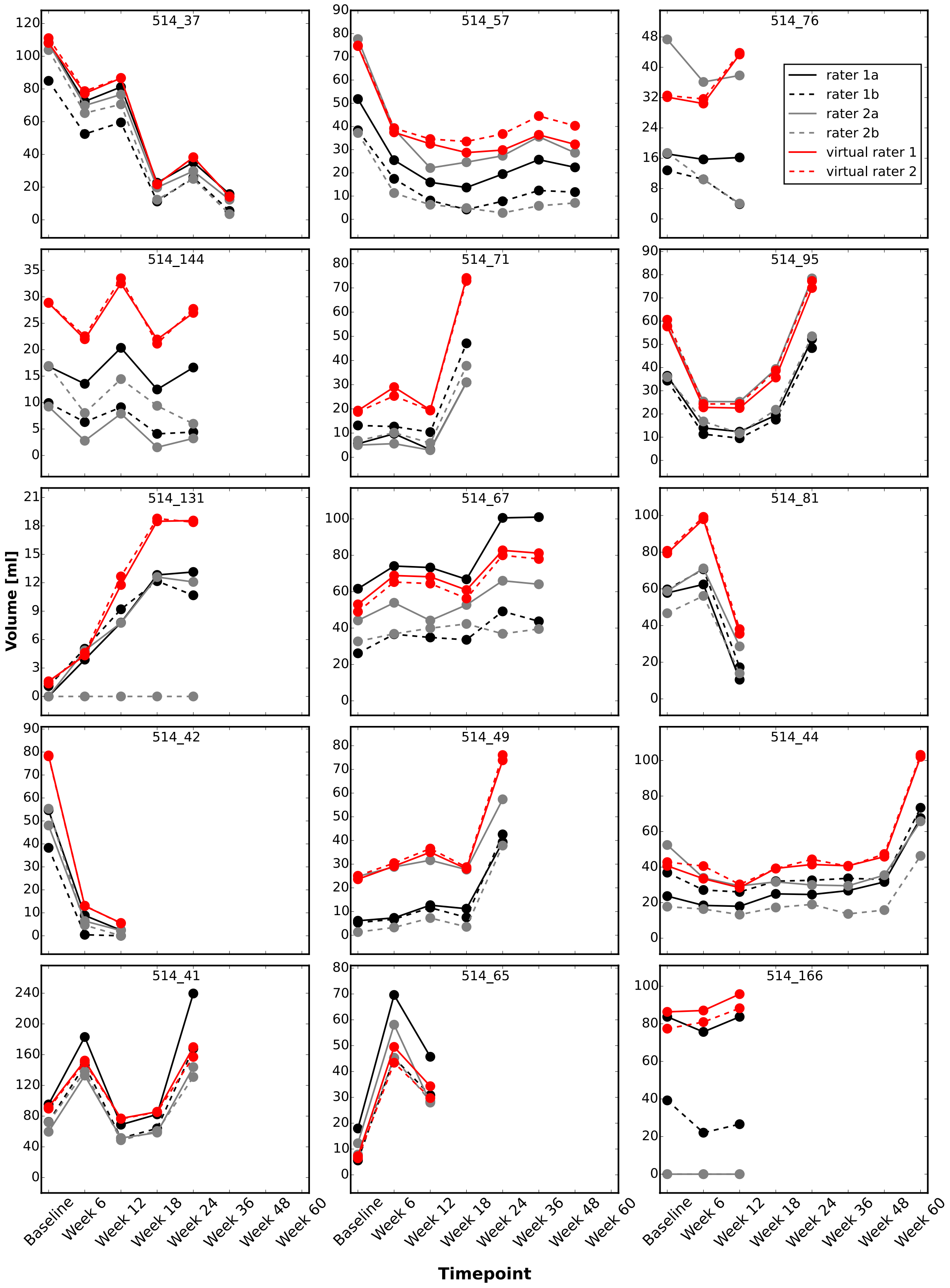

Figure S3b: Longitudinal tumor edema volume for 15 patients suffering from GB. The two human raters interactively segmented the tumor images twice (two independent sessions a and b). The virtual raters show a higher agreement amongst each other but in principle meet the assessments of the human experts.

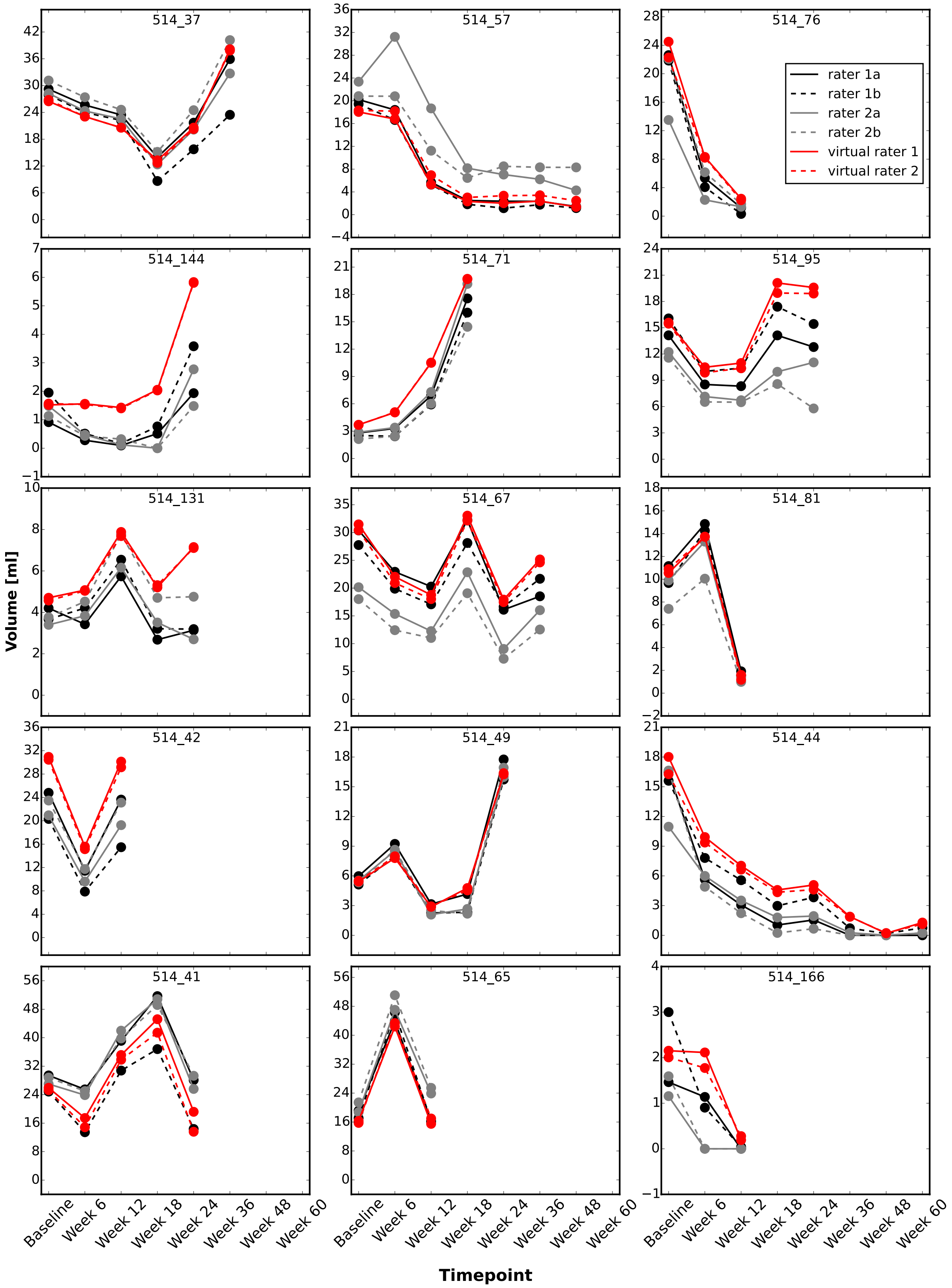

Figure S3c: Longitudinal contrast-enhancing tumor volume for 15 patients suffering from GB. The two human raters interactively segmented the tumor images twice (two independent sessions a and b). The virtual raters show a higher agreement amongst each other but in principle meet the assessments of the human experts.

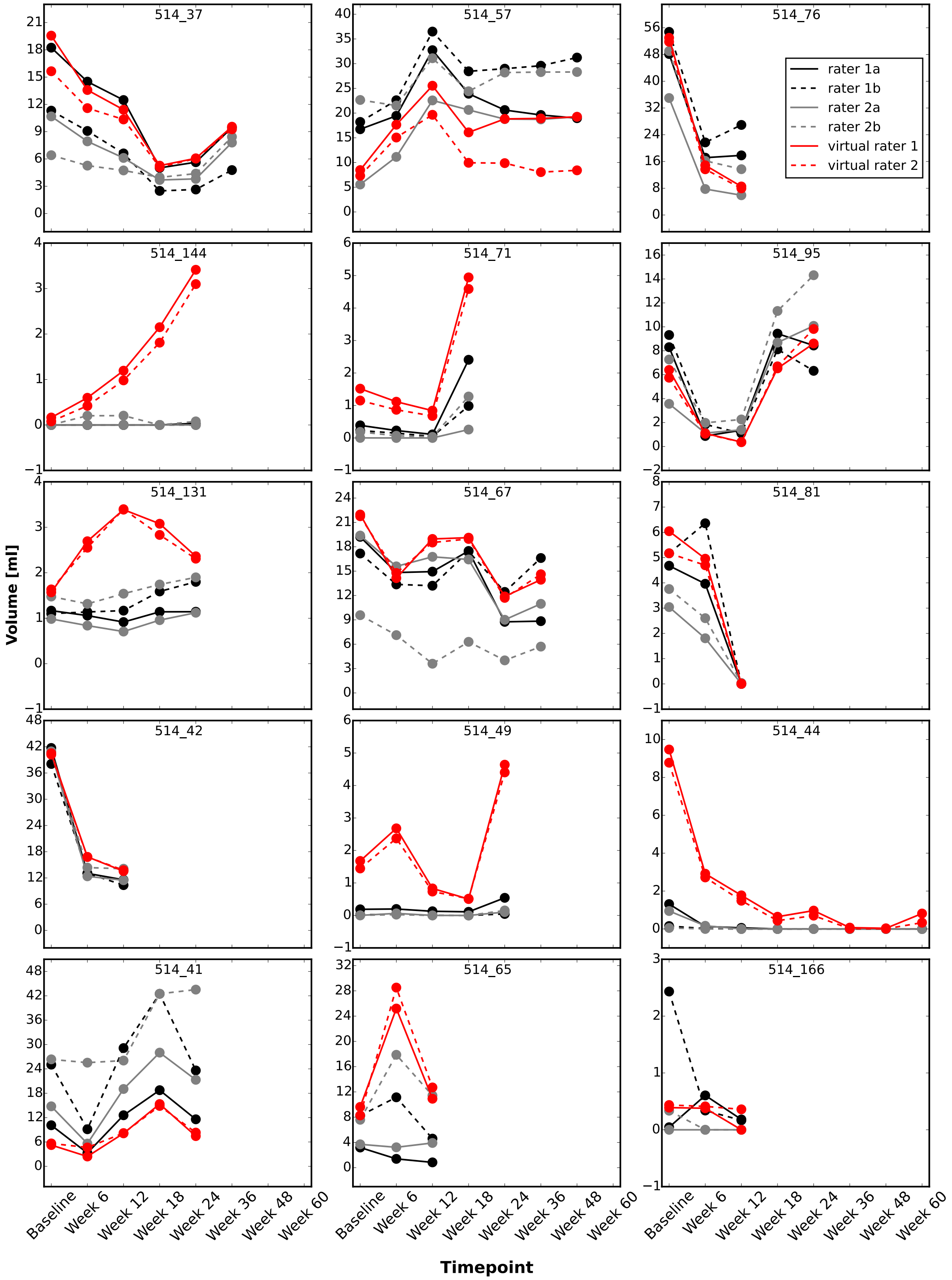

Figure S3d: Longitudinal non-enhancing tumor volume for 15 patients suffering from GB. The two human raters interactively segmented the tumor images twice (two independent sessions a and b). The virtual raters show a higher agreement amongst each other but in principle meet the assessments of the human experts.

Supplementary Tables

Table S1 – Labeling Duration and Tumor Volume Rater 1

Dataset ID	Time Points	Combined Tumor Volume [ml] of all Time Points	Average Tumor Volume [ml]	Duration [s]	Average per Time Point [s]
541_37	6	398	66	1061	177
541_41	5	752	150	1103	221
514_42	3	144	48	519	173
514_44	8	332	42	1254	157
514_49	5	107	21	1046	209
514_57	7	343	49	708	101
514_65	3	185	62	703	235
514_67	6	445	74	1148	191
514_71	4	112	28	1079	267
514_76	3	157	52	856	285
514_81	3	185	62	752	251
514_95	5	217	43	1129	226
514_131	5	66	13	1299	260
514_144	5	41	8	1121	224
514_166	3	95	32	835	278

Table S2 – Label Statistics

Type	Rater	Trial	Category					
			Contrast-Enhancing	Non-Enhancing/ Core	T2 Edema	CSF	Rest	Air
# of Voxels	1	a total	1503	860	2643	1217	2725	1141
		b total	585	689	1139	938	1372	1431
		a avg. (SD)	21 (20)	12 (20)	37 (36)	17 (18)	38 (44)	16 (27)
		b avg. (SD)	8 (12)	10 (20)	16 (20)	13 (22)	19 (32)	20 (38)
		Combined avg.	15	11	27	15	29	18
	2	a total	792	521	1436	1017	1540	1788
		b total	437	334	828	756	1179	1632
		a avg. (SD)	11 (16)	7 (14)	20 (34)	14 (26)	22 (30)	25 (48)
		b avg. (SD)	6 (9)	5 (10)	12 (24)	11 (19)	17 (29)	23 (46)
		Combined avg.	9	6	16	12	19	24
Brush Strokes	1	a total	147	89	156	108	340	61
		b total	143	88	108	114	236	49
		a avg. (SD)	2 (2)	1 (1)	2 (1)	2 (1)	5 (4)	1 (1)
		b avg. (SD)	2 (2)	1 (1)	2 (2)	2 (2)	3 (4)	1 (1)
		Combined avg.	2	1	2	2	4	1
	2	a total	361	177	186	127	798	72
		b total	317	138	97	80	529	60
		a avg. (SD)	5 (5)	2 (4)	3 (4)	2 (3)	11 (11)	1 (2)
		b avg. (SD)	4 (4)	2 (3)	1 (2)	1 (2)	7 (9)	1 (2)
		Combined avg.	5	2	2	1	9	1
Stroke Length	1	a avg.	10.2	9.6	16.9	11.3	8.0	18.7
		b avg.	4.1	7.8	10.5	8.2	5.8	29.2
		Combined avg.	7.2	8.8	14.3	9.7	7.1	23.4
	2	a avg.	2.2	2.9	7.7	8.0	1.9	24.8
		b avg.	1.4	2.4	8.5	9.5	2.3	27.2
		Combined avg.	1.8	2.7	8.0	8.6	2.0	25.9

Table S3 – Leave-one-out cross validation of Dice scores with 1σ standard deviation. Categories that differ significantly ($p<0.01$) according to Welch's Two Sample t-test are denoted with an asterisk (*).

	Category						
	**Gross Tumor**	**Contrast-Enhancing**	**Non-Enhancing/Core***	**T2 Edema**	**CSF***	**Rest**	**Air**
**Human Raters**	0.635 (0.191)	0.528 (0.228)	0.393 (0.290)	0.488 (0.245)	0.547 (0.188)	0.950 (0.045)	0.961 (0.102)
**Virtual Raters**	0.636 (0.166)	0.515 (0.200)	0.281 (0.249)	0.486 (0.197)	0.463 (0.192)	0.958 (0.022)	0.968 (0.020)

Table S4 – Welch's Two Sample t-test for comparison of GTV Dice Scores (BraTS data)

	Human Rater 1 vs. Human Rater 2
Virtual Rater 1 vs. Virtual Rater 2	t(35)=9.4, p << 0.00001

	Human Rater 1 vs. Human Rater 3
Virtual Rater 1 vs. Virtual Rater 3	t(33)=11.9, p << 0.00001

	Human Rater 1 vs. Human Rater 4
Virtual Rater 1 vs. Virtual Rater 4	t(33)=12.1, p << 0.00001

	Human Rater 2 vs. Human Rater 3
Virtual Rater 2 vs. Virtual Rater 3	t(33)=10.4, p << 0.00001

	Human Rater 2 vs. Human Rater 4
Virtual Rater 2 vs. Virtual Rater 4	t(35)=10.5, p << 0.00001

	Human Rater 3 vs. Human Rater 4
Virtual Rater 3 vs. Virtual Rater 4	t(33)=9.7, p << 0.00001

Table S5 – Mean inter-rater Dice scores with 1σ standard deviation

	Category						
	**Gross Tumor**	**Normal**	**Necrosis**	**Edema**	**Non-enhancing tumor**	**Enhancing tumor**	**Air**
**Human Raters**	0.825 (0.069)	0.990 (0.008)	0.586 (0.303)	0.588 (0.280)	0.246 (0.303)	0.651 (0.286)	>0.999 (0.001)
**Virtual Raters P=1.0**	0.963 (0.043)	0.998 (0.002)	0.743 (0.236)	0.922 (0.077)	0.685 (0.190)	0.825 (0.233)	>0.99 (0.001)
**Virtual Raters P=0.75**	0.958 (0.053)	0.997 (0.002)	0.787 (0.200)	0.914 (0.090)	0.648 (0.219)	0.816 (0.252)	0.999 (0.001)
**Virtual Raters P=0.5**	0.956 (0.060)	0.997 (0.002)	0.768 (0.235)	0.909 (0.091)	0.572 (0.268)	0.794 (0.265)	0.999 (0.002)
**Virtual Raters P=0.25**	0.949 (0.078)	0.997 (0.002)	0.726 (0.278)	0.897 (0.106)	0.497 (0.290)	0.797 (0.265)	0.999 (0.002)

Table S6 – Mean Dice scores compared to ground truth (reference segmentation) with 1σ standard deviation

	**Category**						
	**Gross Tumor**	**Normal**	**Necrosis**	**Edema**	**Non-enhancing tumor**	**Enhancing tumor**	**Air**
**Human Raters**	0.899 (0.047)	0.994 (0.005)	0.726 (0.285)	0.746 (0.235)	0.431 (0.382)	0.741 (0.306)	>0.999 (0.001)
**Virtual Raters P=1.0**	0.817 (0.097)	0.978 (0.010)	0.446 (0.313)	0.689 (0.149)	0.296 (0.260)	0.599 (0.337)	0.997 (0.002)
**Virtual Raters P=0.75**	0.805 (0.109)	0.978 (0.009)	0.449 (0.321)	0.693 (0.157)	0.274 (0.263)	0.608 (0.333)	0.997 (0.002)
**Virtual Raters P=0.5**	0.809 (0.108)	0.978 (0.010)	0.452 (0.319)	0.691 (0.155)	0.277 (0.269)	0.596 (0.331)	0.997 (0.002)
**Virtual Raters P=0.25**	0.788 (0.168)	0.976 (0.010)	0.445 (0.319)	0.692 (0.162)	0.272 (0.289)	0.577 (0.343)	0.996 (0.003)

Video Caption

Video 1: Demonstration of the MRIVolumetry Workflow. The exemplary use case illustrates a longitudinal brain tumor examination for a GB patient, including loading of 5D (time, x, y, z, channel) MRI-data, interactive annotation, filtering and report generation. The underlying machine learning algorithm captures the knowledge of the rater in close to real time.
